# Simulation of Continuous Dynamic Recrystallization Using a Level-Set Method

**DOI:** 10.3390/ma15238547

**Published:** 2022-11-30

**Authors:** Victor Grand, Baptiste Flipon, Alexis Gaillac, Marc Bernacki

**Affiliations:** 1 Framatome—Components Research Center, 73403 Ugine, France; 2 Mines Paris, PSL University, Centre for Material Forming (CEMEF), UMR CNRS, 06904 Sophia Antipolis, France

**Keywords:** level-set, grain growth, continuous dynamic recrystallization, numerical simulation, hot forming

## Abstract

Dynamic recrystallization is one of the main phenomena responsible for microstructure evolution during hot forming. Consequently, obtaining a better understanding of dynamic recrystallization mechanisms and being able to predict them is crucial. This paper proposes a full-field numerical framework to predict the evolution of subgrain structures upon grain growth, continuous dynamic recrystallization, and post-dynamic recrystallization. To be able to consider a subgrain structure, two strategies are proposed. One relies on a two-step tessellation algorithm to generate a fully substructured microstructure. The second strategy enables for the simulation of the formation of new subgrains during hot deformation. Using these tools, the grain growth of a fully substructured microstructure is modeled. The influence of microstructure topology, subgrain parameters, and some remaining stored energy due to plastic deformation is discussed. The results highlight that the selective growth of a limited number of subgrains is observed only when mobility is a sigmoidal function of disorientation. The recrystallization kinetics predicted with different criteria for discrimination of recrystallized grains are quantitatively compared. Finally, the ability of the framework to model continuous dynamic and post-dynamic recrystallization is assessed upon a case study representative of the hot extrusion of a zircaloy-4 billet (T=650 °C$;\;\dot{\varepsilon }=1.0\;s^{-1};\;\varepsilon_{f}=1.35$). The influence of grain boundary properties and nucleation rules are quantified to evaluate the model sensitivity and suitability. Application of these numerical tools to other thermomechanical conditions and microstructures will be presented in an upcoming article.

## 1. Introduction

Dynamic recrystallization (DRX) is one of the main phenomena responsible for microstructure evolution during the hot forming operations of metallic materials. Improving our ability to model those mechanisms is of critical interest because it would allow for an assessment of the influence of material and processing parameters and a reduction in the number of experiments required to optimize industrial manufacturing paths [1]. DRX is defined as the formation of new grains with low dislocation density that progressively consume the deformed microstructure under hot deformation conditions [1,2]. However, this mechanism of microstructure evolution can exhibit various typical features. Therefore, based on those characteristics, DRX is commonly classified into three categories [3]:

- Discontinuous DRX (DDRX), if recrystallized grains nucleate at some specific locations, generally close to grain boundaries (GB), and then grow and consume the deformed grains surrounding them. DDRX is therefore characterized by spatial and temporal discontinuity at the polycrystal scale.
- Continuous DRX (CDRX), when recrystallized grains form slowly and continuously during deformation. In that case, grain formation is induced by the progressive reorganization of dislocations into cells or subgrains and the gradual increase in misorientation angle between those subgrains.
- Geometric DRX (GDRX), at large strains when grains become serrated and some GBs start to meet and enclose new grains.

It should be pointed out that, depending on the material and on the thermomechanical conditions, classifying a mechanism as discontinuous or continuous is not straightforward [1]. CDRX and DDRX are based on the same physical phenomena taking place at different spatial and temporal scales which leads to the different features mentioned previously. The predominance of one or the other mechanism is influenced by both material characteristics and thermomechanical conditions. DDRX is known to happen in low to medium stacking fault energy (SFE) materials whereas CDRX is mostly found in high SFE metals [4]. This is explained by the fact that low SFE materials exhibit much less dynamic recovery (DRV) and are less ready to form low-angle grain boundaries (GBs that have a disorientation lower than a given threshold, often fixed to $15^{{}^{\circ}}$ and denoted as LAGB) or substructures as a result. Regarding processing parameters, it has been found that low strain rates tend to favor CDRX [1].

Until now, most of the research efforts at the mesoscopic scale have been applied to observe, characterize, and model DDRX. This appears natural, because common study materials such as stainless steels and nickel-based superalloys undergo these mechanisms under a usual range of thermomechanical parameters [5,6]. In addition, it should be mentioned that modeling DDRX at the mesoscale is more evident because nucleation of recrystallized grains can be implemented naturally within common simulation frameworks [7], whereas modeling CDRX requires the ability to describe substructure formation and evolution. Therefore, one can easily understand why the majority of numerical studies focus on DDRX [8,9,10].

Recently, several articles focusing on the physical mechanisms underlying CDRX have been published [11,12,13]. They provide an in-depth characterization of CDRX for different alloys and provide some new insight upon substructure formation and evolution. Regarding simulation of CDRX, Gourdet et al. [14] published one of the first articles on this topic. They proposed a mean-field model, i.e., a model that considers the average main microstructure characteristics and does not directly describe the microstructure topology. They applied this model to simulate the CDRX of aluminum alloys. Their model relies on a schematic representation of the microstructure as an aggregate of grains and subgrains. LAGB are grouped into classes of same orientation that undergo a progressive increase in disorientation. If sufficient deformation is applied, these LAGB could possibly transform into high-angle grain boundaries (HAGB, i.e., GB that have a disorientation higher than $15^{{}^{\circ}}$) and contribute to form recrystallized grains. This work still inspires many researchers and some recent articles illustrate attempts at expanding it. These publications especially highlight that additional phenomena can be considered, such as post-dynamic recrystallization (PDRX) [15], precipitate–dislocation interactions [16], and saturation of the average subgrain disorientation [17]. These studies [15,16,17] confirmed the ability of the Gourdet–Montheillet model to simulate CDRX. Nevertheless, the models used within these studies suffer from the intrinsic limitations of mean-field approximations, which makes consideration of microstructure heterogeneities impossible.

The article presents a full-field model of CDRX that offers the possibility to assess the influence of fine microstructural features upon CDRX. An extension of a DDRX modeling framework [10,18] is proposed to simulate CDRX. It relies on a level-set (LS) formulation to simulate microstructure evolution during heat treatment and hot deformation. It takes advantage of recent developments regarding simulation of anisotropic grain growth [19,20] to describe precise kinetic substructures. It is applied to 2D simulations because an extensive comparison to 2D experimental results will be provided in an upcoming study. Two strategies are employed to introduce substructures. In grain growth (GG) simulations, fully substructured microstructures are initially generated and their migrations are modeled. In dynamic and post-dynamic simulations, the formation and evolution of subgrains under deformation is tackled by implementing mean-field equations into the LS framework. With these modifications, this paper presents the first numerical framework that is able to model DDRX and CDRX with a high degree of consistency between the physical phenomena considered, the numerical strategies and the material parameters.

The article first introduces the numerical framework, including the basic theory underlying the LS method. Then, generation of digital microstructures and the physical phenomena considered are detailed. The following sections are devoted to the presentation of the simulation results for GG, CDRX, and PDRX. The influences of specific microstructure characteristics are pointed out and discussed in relation to the results available in the recent literature.

## 2. Numerical Framework

### 2.1. Grain Boundary Description

The model used in this paper describes GB using an LS formulation. The iso-zero values of level-set functions ϕ are used to track microstructure interfaces in space and time. LS functions are initialized according to Equation (1):

(1) $$\begin{array}{c} \left\{\begin{array}{c} \phi \left(x,t\right)=\pm \;d\left(x,\Gamma (t)\right),\;x\in \Omega ,\\ \Gamma (t)=\{x\in \Omega ,\;\phi (x,t)=0\}, \end{array}\right. \end{array}$$

where *d* is the signed Euclidean distance function and Ω is the simulation domain. ϕ(x,t) is a positive value inside the grain and a negative value outside. Initially, one LS function was defined per grain. To reduce the number of functions, a graph coloring strategy was applied [21]. We can denote $\Phi =\{\phi_{i},\;i=1,\;\ldots ,\;N\}$ as the set of all distance functions used to describe all the grains. *N* is the number of distance functions and is significantly smaller than the number of grains ($N_{g}$).

The movement of interfaces is described by solving, for each considered distance function, the following transport equation:

(2) $$\begin{array}{c} \frac{\partial \phi }{\partial t}+\vec{v}\cdot \vec{\nabla \phi }=0, \end{array}$$

with $\vec{v}$ being the velocity of interfaces. At the mesoscopic scale, $\vec{v}$ is generally expressed as the sum of a capillarity term (${\vec{v}}_{c}$) and of a second one induced by stored energy due to plastic deformation (${\vec{v}}_{e}$). These two terms are defined such as [1,18]:

(3) $$\begin{array}{c} {\vec{v}}_{c}=-M\gamma \kappa \vec{n}, \end{array}$$

(4) $$\begin{array}{c} {\vec{v}}_{e}=M〚E〛\vec{n}, \end{array}$$

where *M*, γ, and κ are the mobility, energy, and trace of the curvature tensor of GB, respectively. 〚E〛 is the stored energy gap between adjacent grains. At the grain boundary vicinity, it can be expressed such as: $〚E\left(x,t\right)〛=f\left(\phi_{i}\left(x,t\right),l\right)\times \left(E_{j}\left(t\right)-E_{i}\left(t\right)\right)$, where f is a decreasing function equal to 1 for $\phi_{i}=0$ to 0 for $\phi_{i}=l$ and $E_{i}\left(t\right)$ and $E_{j}\left(t\right)$ are the stored energy for grains i and j, respectively. Finally, $\vec{n}$ is the outward unitary normal of GB. The detailed procedure of the computation of ${\vec{v}}_{e}$ is presented in ref. [22]. Stored energy is computed using a dislocation density field:

(5) $$\begin{array}{c} E=\tau \rho \end{array}$$

τ is the unit dislocation line energy and is defined as a material parameter. Its value is computed with the equation $\tau =\frac{\mu b^{2}}{2}$, where μ is the shear modulus and *b* is the Burgers vector [23]. This field is averaged per grain/subgrain. During simulation, the dislocation density of the surface swept by each interface is reset to a low value ($\rho_{0}$) and the energy is averaged again. Therefore, the stored energy decreases in grains/subgrains that grow, whereas it stays constant for the ones shrinking.

κ and $\vec{n}$ can be defined naturally by taking advantages of the possibilities offered by the LS framework. Indeed, considering that LS functions remain as distance functions all along the simulation (i.e., that $∥\vec{\nabla \phi }∥=1$), they can be defined as:

(6) $$\begin{array}{c} \vec{n}=-\vec{\nabla \phi }, \end{array}$$

(7) $$\begin{array}{c} \kappa =-\Delta \phi . \end{array}$$

*M* and γ are material parameters that should be identified carefully. The setting of those parameters as well as other initial microstructure descriptors (such as initial stored energy) is described in detail in Section 2.3.

Because CDRX involves a significant presence of LAGBs, being able to predict their evolution is crucial. To perform this, the evolution of properties with misorientation must be described properly. In the simulations presented in this work, γ is always considered as heterogeneous (i.e., γ being a function of disorientation, γ=γ(θ)) and *M* is either considered homogeneous or heterogeneous, depending on cases. This induces the introduction of an additional term inside the velocity Equation (Equation (3)) [19,20,24], such that:

(8) $$\begin{array}{c} {\vec{v}}_{c}=-M\left(\vec{\nabla \gamma }\cdot \vec{n}-\gamma \kappa \right)\vec{n}, \end{array}$$

which, using Equations (6) and (7) becomes:

(9) $$\begin{array}{c} {\vec{v}}_{c}=-M\left[\left(\vec{\nabla \gamma }\cdot \vec{\nabla \phi }\right)\vec{\nabla \phi }-\gamma \Delta \phi \vec{\nabla \phi }\right]. \end{array}$$

Finally, the transport equation becomes:

(10) $$\begin{array}{c} \frac{\partial \phi }{\partial t}+{\vec{v}}_{e}\cdot \vec{\nabla \phi }+M\left[\vec{\nabla \gamma }\cdot \vec{\nabla \phi }-\gamma \Delta \phi \right]=0. \end{array}$$

The weak formulation of the previous equation, with $\varphi \in H_{0}^{1}\left(\Omega \right)$ is defined as:

(11) $$\begin{array}{c} \begin{array}{c} \int_{\Omega }\frac{\partial \phi }{\partial t}\varphi d\Omega +\int_{\Omega }M\gamma \vec{\nabla \varphi }\cdot \vec{\nabla \phi }d\Omega -\int_{\Omega }{\vec{v}}_{e}\cdot \vec{\nabla \phi }\varphi d\Omega \\ +2\int_{\Omega }M\vec{\nabla \gamma }\cdot \vec{\nabla \phi }\varphi d\Omega +\int_{\Omega }\gamma \vec{\nabla M}\cdot \vec{\nabla \phi }\varphi d\Omega \\ -\int_{\partial \Omega }M\gamma \varphi \vec{\nabla \phi }\cdot \vec{n_{\partial \Omega }}d\left(\partial \Omega \right)=0. \end{array} \end{array}$$

*M* and γ are by definition highly discontinuous because they present positive values in elements crossed by GB and are null in all other elements. To avoid numerical issues during computation of $\vec{\nabla \gamma }$ and $\vec{\nabla M}$, a Laplace equation is solved to obtain first order differentiable fields with the correct values at GB [19].

This formulation is not the most complete available at the time, because some others are able to consider anisotropic grain properties, e.g., that also depend on the normal GB [25]. However, according to the conclusions of the study made by Murgas et al. [20], this heterogeneous formulation remains a good compromise between computation costs and prediction of GB kinetics and morphology.

### 2.2. Generation and Evolution of Microstructures

#### 2.2.1. Generation of A Two-Level Microstructure

A first method to obtain a microstructure exhibiting substructures is to employ a two-level generation method. The generation strategy relies on a Laguerre–Voronoï algorithm [26] using an optimized sphere packing strategy [27]. This generation algorithm is called twice. First, it is used to generate the grain structure in a standard way. Then, it is called again to generate the subgrain structure. The nuclei of the Voronoï cells located too close to the initial GB are removed. Then, LS functions are defined based on those cells.

**Figure 1:**
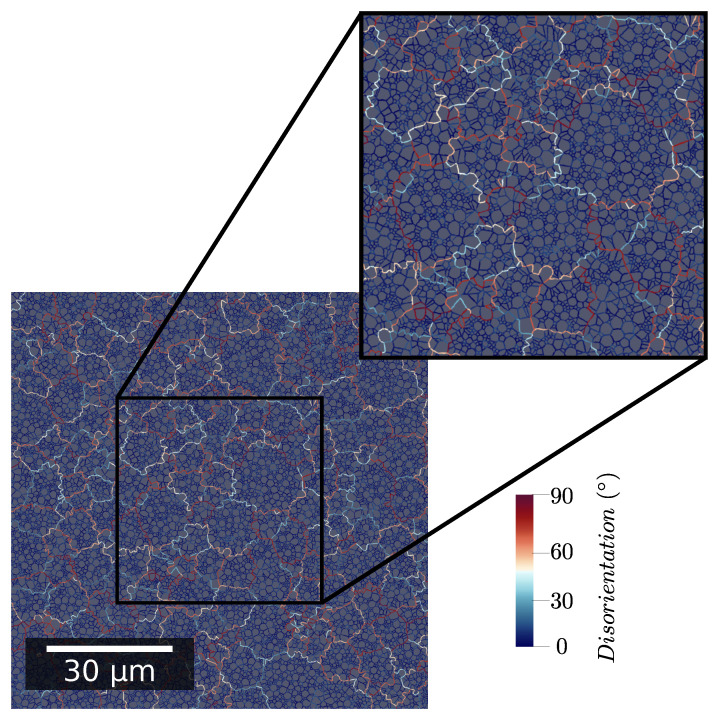
Initial microstructure example with color code corresponding to GB misorientation angle.

The generation algorithm is in charge of respecting the prescribed grain and subgrain size distributions. Grain orientations are affected in order to respect a given distribution taken from experimental data obtained on a zircaloy-4 sample. Subgrain orientation is computed by applying a specific misorientation to the parent grain orientation, similarly to what has been achieved in ref. [28]. This misorientation distribution is also taken from experimental data. Finally, an image of an initial microstructure is shown in Figure 1. The zone at the center that is enlarged is kept the same in Figure 5 to improve the readability.

It is worth noting that this method allows for the generation of various initial configurations and detailed study of the influence of topology. As illustrated in Section 3.1, this enables the enrichment of the rare existing discussions about the full-field modeling of the evolution of subgrain structures during grain growth [28,29,30].

#### 2.2.2. Progressive Formation of Subgrains

As described in the introduction (Section 1), under deformation, the LAGB network does not evolve only by capillarity growth. Dislocations could rearrange themselves to form new LAGBs or accumulate into preexisting LAGBs. This last phenomenon is responsible for a progressive increase in LAGB misorientation [1,3]. To be able to consider these mechanisms, laws introduced by the Gourdet–Montheillet model are implemented [14].

Each grain has an average dislocation density ρ which evolves according to the Yoshie–Laasraoui–Jonas Equation [31]:

(12) $$\begin{array}{c} d\rho =\left(K_{1}-K_{2}\rho \right)d\varepsilon , \end{array}$$

where $K_{1}$ and $K_{2}$ are two material constants describing the strain hardening and recovery, respectively. To be able to reproduce to some extent heterogeneous grain deformation, $K_{1}$ varies from grain to grain according to a distribution defined using experimental data (see Section 2.3). This strategy is preferred over the coupling with a crystal plasticity model because it would unreasonably increase the computational costs. Several mechanisms of dislocation density evolution are taken into account in the current model:

- Rearrangement into LAGB that bound new subgrains. Subgrain formation is described through following Equation [14]:
  (13) $$\begin{array}{c} dS^{+}=\frac{\alpha bK_{2}\rho d\varepsilon }{\eta \theta_{0}}, \end{array}$$
  where $dS^{+}$ is the surface of LAGB created. $\alpha =1-\exp{\left(\frac{D}{D_{0}}\right)}^{m}$ is a coefficient describing the fraction of dislocations recovered to form new subgrains. *D* is the grain diameter, $D_{0}$ is a grain reference diameter, and *m* is a fixed coefficient. η is the number of sets of dislocations and $\theta_{0}$ the disorientation of newly formed subgrains.
- Stacking into pre-existing LAGBs, which is modeled according to the following Equation [14]:
  (14) $$\begin{array}{c} d\theta =\frac{b}{2\eta }\left(1-\alpha \right)DK_{2}\rho d\varepsilon . \end{array}$$
- Absorption during HAGB migration. This is naturally captured by affecting the areas swept by moving boundaries with a low dislocation density as described earlier.

At each time step, dislocation density of each grain is updated using Equation (12) which impacts the computation of the velocity term related to the stored energy differences. Then, the length of subgrain interfaces formed into each grain is computed using Equation (13). Depending on the simulation case (see Section 3.2), subgrains are added grain by grain based on the value of the grain property or globally, after having summed the length of subgrain interfaces for all grains. Subgrain orientation is initialized by applying a small misorientation to the parent grain orientation (similarly to what is described in Section 2.2.1). The misorientation angle is selected to respect a distribution measured experimentally, whereas the misorientation axis satisfies a uniform distribution. The misorientation axis attributed to a subgrain at its formation is kept constant. Then, during the next increments, the misorientation increase described by Equation (14) is realized by rotating of dθ around this axis. In addition, to evaluate the influence of subgrain formation localization, a distance from the pre-existing boundaries criterion is tested. Therefore, for the cases where this option is enabled, subgrain centers cannot be set closer to pre-existing interfaces than $d_{\mathrm{SG}}^{f}$. This will be discussed in more details in Section 3.2.

**Figure A2:**
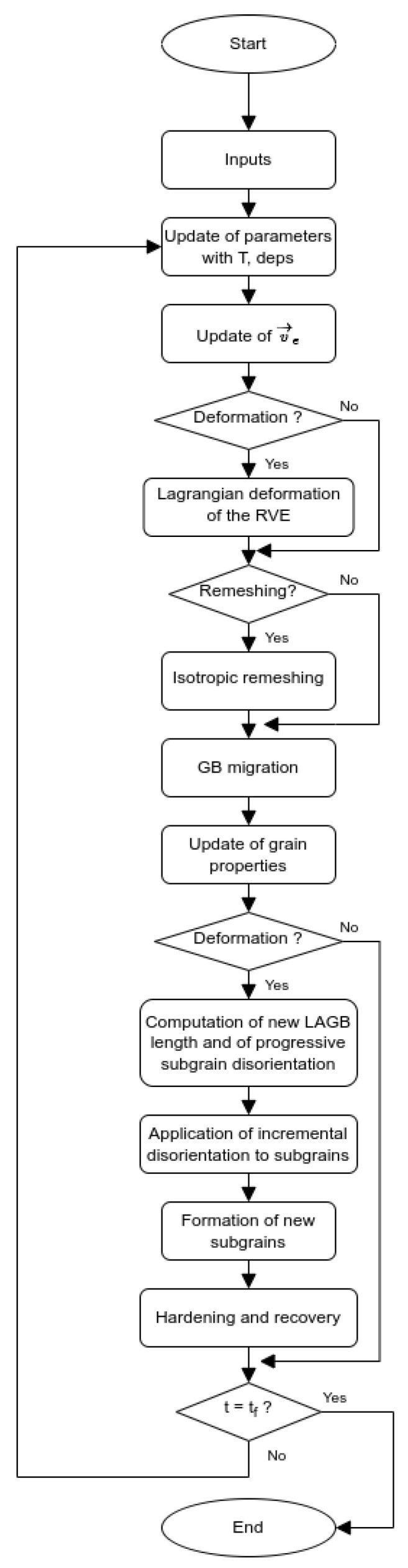
Algorithm describing the numerical steps to simulate microstructure evolutions during hot deformation and holding at temperature.

Finally, at each time step, isotropic remeshing is performed to keep a fine mesh around GB and precise description of interfaces [32,33]. All those operations are presented in an algorithmic diagram in Figure A2.

### 2.3. Material Parameters

GB energy is defined according to Read–Schockley Equation [34]:

(15) $$\begin{array}{c} \gamma \left(\theta \right)=\left\{\begin{array}{c} \gamma_{max}\left(\frac{\theta }{\theta_{max}}\right)\left(1-\ln\frac{\theta }{\theta_{max}}\right),\;\theta <\theta_{max},\\ \gamma_{max},\;\theta \geq \theta_{max}, \end{array}\right. \end{array}$$

where $\theta_{max}$ is the limit between LAGB and HAGB, taken equal to $15^{{}^{\circ}}$.

If not explicitly written, GB mobility, *M*, is considered isotropic and does not vary with disorientation (i.e., $M=M_{max}$). HAGB mobility ($M_{max}$) is identified using experimental data. Its dependence to temperature is supposed to follow Arrhenius’ law. In some given simulation cases and to enable the discussion and comparison of the results to the ones described by Suwa et al. [30], mobility is described by the following relation [35]:

(16) $$\begin{array}{c} M\left(\theta \right)=\left\{\begin{array}{c} M_{max}\left(1-\exp\left[-5{\left(\frac{\theta }{\theta_{max}}\right)}^{4}\right]\right),\\ M_{max},\;\theta \geq \theta_{max}. \end{array}\right. \end{array}$$

## 3. Results and Discussion

### 3.1. Modeling of GG of a Fully Substructured Microstructure

#### 3.1.1. Influence of Microstructure Topology

**Figure 2:**
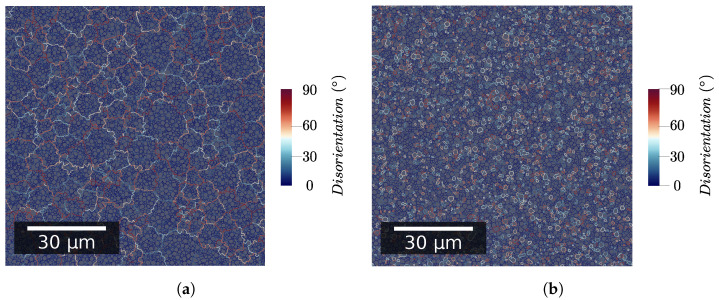
Initial GB network with color code corresponding to GB disorientation. (**a**) Standard grain/subgrain topology; (**b**) No grain/subgrain topology.

To evaluate the influence of microstructure topology, two different initial microstructures are considered:

- With subgrains located inside grains using the generation method described in Section 2.2.1 (Figure 2a);
- With LAGB and HAGB evenly distributed throughout the whole representative volume element (RVE) (see Figure 2b).

The first microstructure topology is named in the following discussions as *standard grain/subgrain topology*. The second is named the *no grain/subgrain topology*. The two initial microstructures are presented in Figure 2.

For all simulation cases presented in this section, GB energy is taken to be heterogeneous, GB mobility is considered isotropic, and stored energy is considered negligible (i.e., $v_{e}=0$). The RVE area is equal to 0.01 mm${}^{2}$.

**Figure 3:**
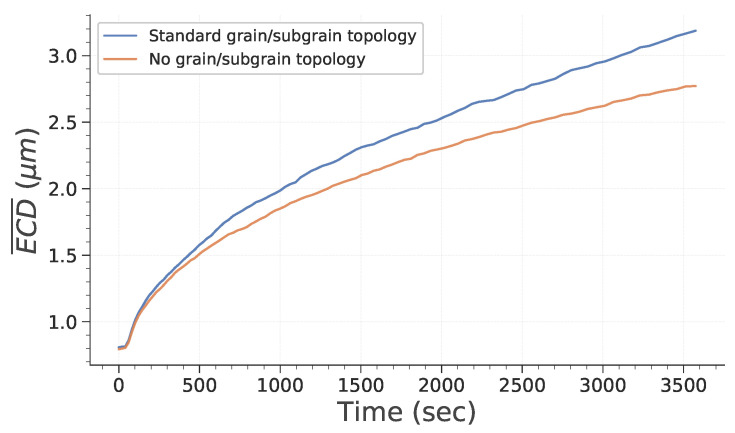
Evolution of subgrain average equivalent circle diameter ($\bar{ECD}$) as a function of time.

**Figure 4:**
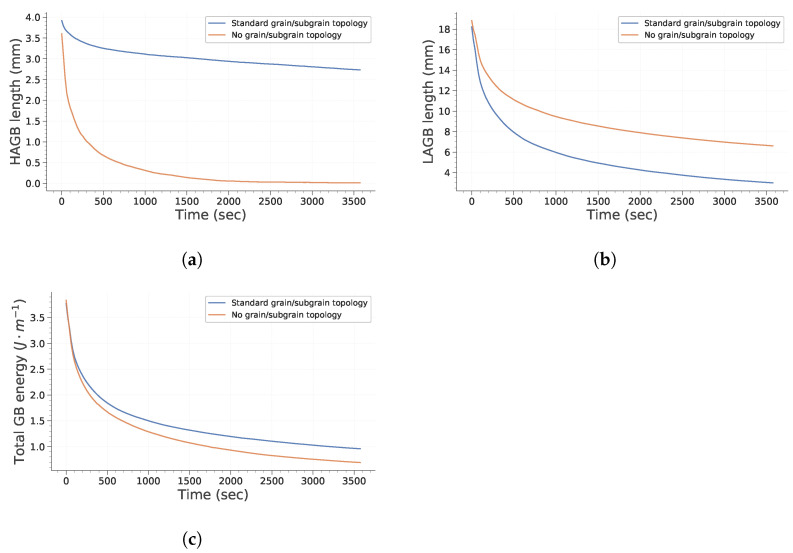
Evolution of GB length and energy with time. (**a**) Total HAGB length; (**b**) Total LAGB length; (**c**) Total GB energy.

Figure 3 and Figure 4 point out how spatial correlation of HAGB and LAGB impacts microstructure evolution. First, it is interesting to note that the absence of spatial correlation decreases the grain growth kinetic. This can be explained partly by the fact that grains (i.e., bounded by HAGB) disappear faster when no correlation is assumed (as illustrated in Figure 4a). Therefore, this leads to a lowering of the global grain growth kinetic because the global system energy is initially lowered much faster. It is interesting to note that this behavior is different from the one expected by Desprès et al. [28]. Two reasons can lead to these differences. First, Desprès et al. [28] consider subgrains partly bounded by HAGB. Second, the initial subgrain distribution is different from our study case, which could also impact the microstructure evolution.

Finally, when considering the influence of spatial distribution of orientations and misorientations, one limitation of this work should be pointed out. Indeed, using the generation method presented earlier, it is not possible to generate subgrains inside a grain presenting a misorientation gradient, a feature which is commonly observed in experiments and which could also impact the microstructure evolutions. This could, for instance, lead to an acceleration of the formation of HAGBs by putting in contact two subgrains with a disorientation greater than $15^{{}^{\circ}}$. Nevertheless, this limitation can be overcome by directly immersing experimental data.

#### 3.1.2. Influence of Subgrain Parameters

To assess the influence of subgrain parameters, two different initial microstructures are considered:

- With grain and subgrain size distributions taken from experimental data;
- With grain distribution taken from experimental data and a unique subgrain size.

For each of these initial microstructures, two cases are considered in which some material parameters differ:

- GB energy is taken heterogeneous and GB mobility is considered constant;
- GB energy and mobility are both considered heterogeneous.

In these cases, the standard grain/subgrain topology is assumed and stored energy is neglected (i.e., $v_{e}=0$).

**Figure 5:**
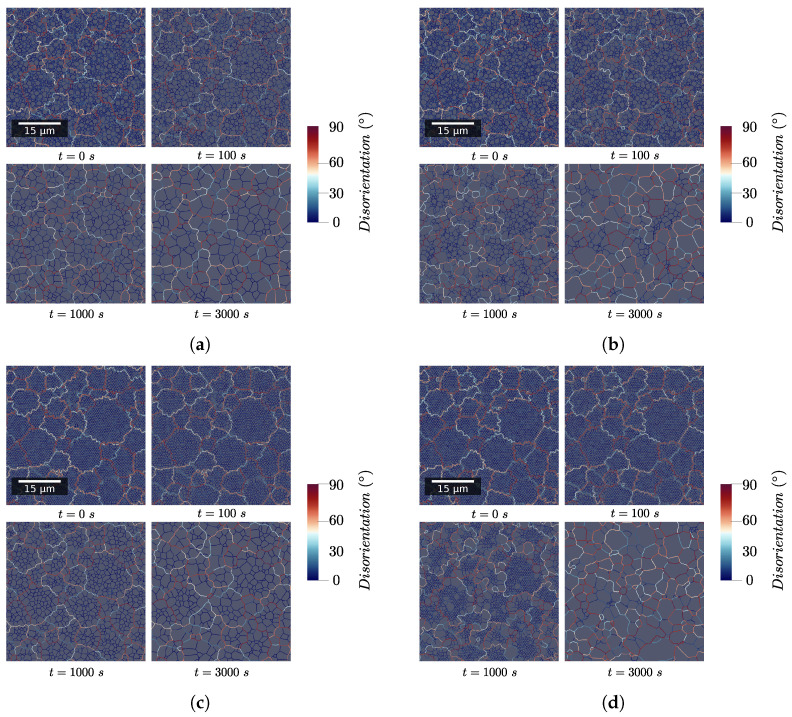
Evolution of the GB network with time for the four test cases in a zone at the center of the RVE. (**a**) Experimental subgrain size distribution—$\gamma_{h}$. (**b**) Experimental subgrain size distribution—$\gamma_{h},\;M_{h}$. (**c**) Uniform subgrain size distribution—$\gamma_{h}$. (**d**) Uniform subgrain size distribution—$\gamma_{h},\;M_{h}$.

Figure 5 presents for each simulation case the GB network in a part of the RVE. Evolution of the whole RVE are available in Appendix A. This illustrates how microstructure evolves in a noticeable manner in case (d).

**Figure 6:**
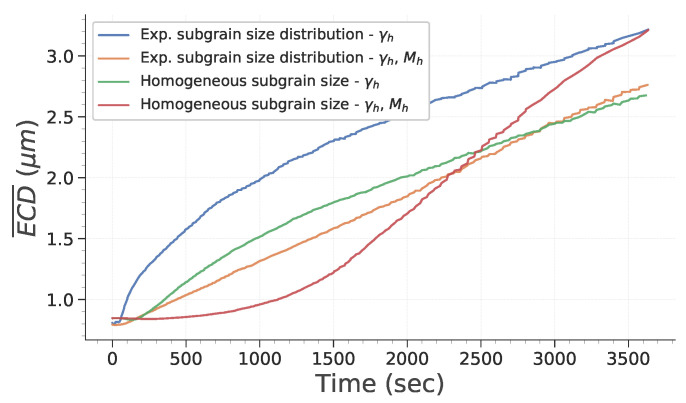
Evolution of subgrain average ECD as a function of time.

**Figure 7:**
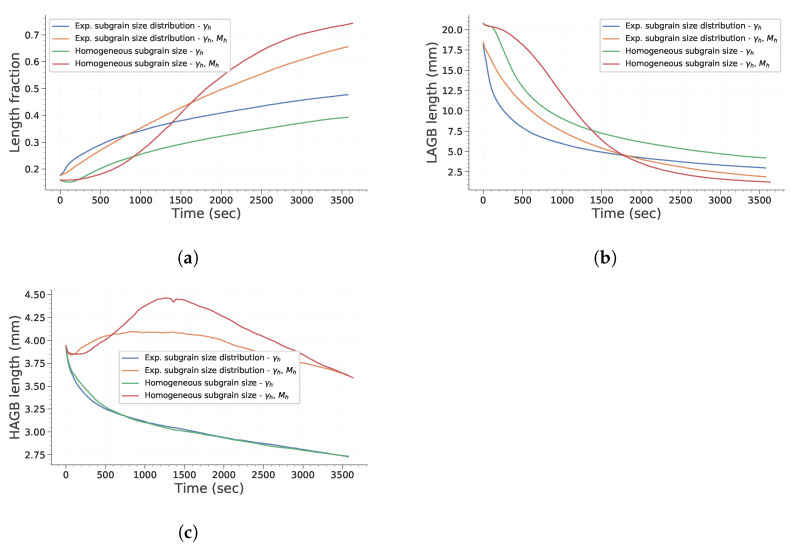
Evolution of GB length with time. (**a**) HAGB length ratio. (**b**) Total LAGB length. (**c**) Total HAGB length.

**Figure 8:**
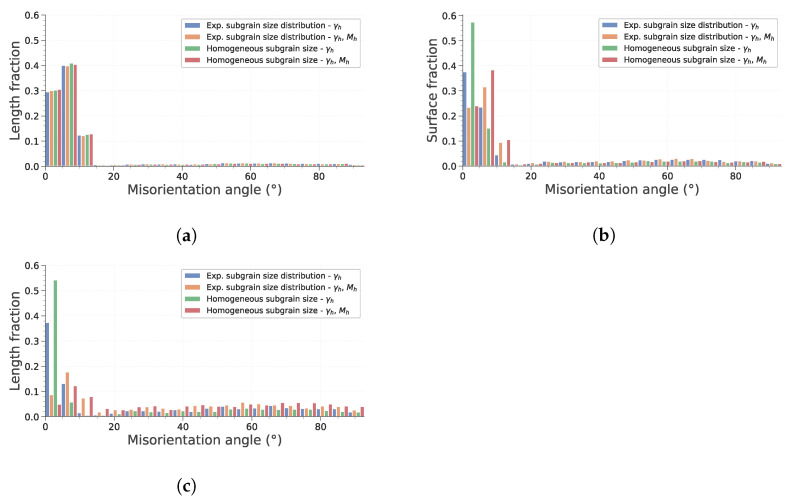
Disorientation histograms at three instances of the simulation. (**a**) Initial state. (**b**) After 1000s. (**c**) Final state.

Figure 6 describes how the mean ECD evolves with time during simulation. First, it is interesting to note that considering heterogeneous mobility (according to Equation (16)) significantly affects subgrain growth and that its impact depends on initial subgrain size distribution. To assess this in more detail, the evolution of total HAGB and LAGB length and of HAGB length fraction are plotted in Figure 7. HAGB length ratio (Figure 7) exhibits how heterogeneous mobility favors the migration of HAGB and their formation by putting in contact subgrains originally located in different grains. At the end of the simulation, this leads to a higher HAGB length fraction. Moreover, as heterogeneous mobility favors HAGB migration, subgrains partially bounded by HAGB have a greater chance to grow. This is particularly true in case *d* of Figure 5, in which all subgrains initially have the same size. Consequently, the advantage given by the high mobility of those boundaries is increased and their growth dominates the whole microstructure evolution. Figure 7b,c confirm these remarks by showing a temporary increase in HAGB total length if mobility is heterogeneous. Finally, it is interesting to note that if subgrains initially have the same diameter, an incubation time is needed for the subgrains partially closed by HAGB to gain a significant advantage and start to consume the whole microstructure. Finally, Figure 8 confirms these observations. It is noteworthy that both study cases assuming homogeneous mobility tend to keep a significantly higher fraction of LAGB. This illustrates that a higher heterogeneity of GB induces a change in the behavior adopted to lower the total energy of the system. It changes from a global reduction in both LAGB and HAGB length to a temporary increase in HAGB length in order to accelerate the decrease in total GB length.

These detailed observations confirm that the faster growth of a small fraction of subgrains is only observable if mobility is considered heterogeneous, as described by Holm et al. [29]. They also broaden the remarks made by Suwa et al. [30] about the conditions allowing preferential subgrain growth. Indeed, this phenomenon is much less significant if the initial subgrain size is not homogeneous and representative of experimental data.

#### 3.1.3. Influence of Stored Energy

In previous papers describing similar full-field simulations, driving pressure due to dislocation density heterogeneities is generally neglected [28,29,30]. This rests upon the simplification that deformation energy is fully consumed thanks to recovery by annihilation and/or organization into the GB network. The available framework allows us to assess the influence of the assumptions by considering that each subgrain has its own dislocation density. Two simulation cases are defined:

- Stored energy is initialized per subgrain by considering a dislocation density distribution taken from estimation of geometrically necessary dislocations (GND) by EBSD measurements.
- Stored energy is initialized per grain using the same distribution. Then, subgrain energy is initialized weighing the parent grain using coefficients (named $w_{i}$) respecting normal distribution. The parameters of this normal distribution are defined as follows: $w\in \left[0.1;2.0\right]$; $\bar{w}=1.0$; $\sigma^{2}=0.2$. The distribution parameters have been set to ensure that subgrains inside grains do not have the exact same energy and that it is still a driving pressure.

**Figure 9:**
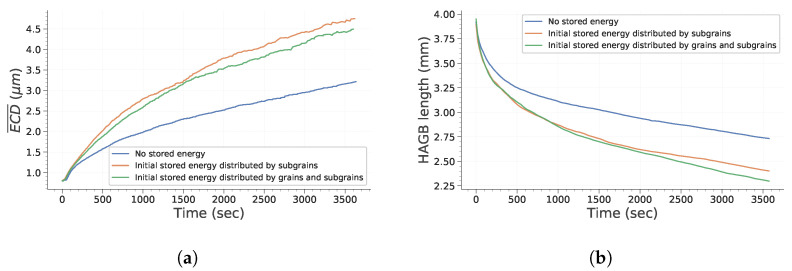
Main microstructural feature evolutions impacted by initial stored energy. (**a**) Evolution of $\bar{ECD}$ as a function of time. (**b**) HAGB total length as a function of time.

Figure 9 presents the main microstructure features that are influenced by the presence of stored energy. As expected, considering stored energy variations between grains leads to an increase in the driving pressures and consequently to a faster growth of subgrains presenting an initial stored energy advantage. Because the total stored energy in the two last simulations is the same, differences in terms of the kinetics are rather small. However, it is interesting to note that in the second case considering stored energy, the main subgrain size evolves slightly slower, whereas the total HAGB length decreases faster. The faster disappearance of HAGB is likely related to the stored energy distribution. Indeed, stored energy gaps between subgrains located at grain interfaces is higher than between subgrains belonging to the same parent grain. This contributes to the acceleration of the movement of HAGB and reduce the growth of subgrains located in inner grain regions.

#### 3.1.4. Discussion of The Numerical Criterion for Identification of Recrystallized Grains

In all of the papers presenting full-field simulations of microstructure evolution including substructures, recrystallized grains are identified based on their size. Subgrains are generally considered as recrystallized grains as soon as they are six to eight times bigger than the initial mean subgrain size [28,29,30]. This relies on the hypothesis that all subgrains are free of energy and could become recrystallized grains. However, in recent experimental studies, recrystallized grains are generally identified based on some parameter quantifying internal disorientation or directly upon GND density [1,36]. To correctly evaluate the recrystallized fraction and to compare the experimental data to numerical data, several criteria are defined and compared. Subgrains are considered as recrystallized if:

- They are eight times bigger than the initial mean subgrain size;
- Their internal dislocation density is lower than a given threshold;
- Their internal dislocation density is lower than a given threshold and at least half of the boundaries surrounding it are HAGBs.

Obviously, the last two criteria are only tested against the simulations that consider stored energy (see Section 3.1.3).

**Figure 10:**
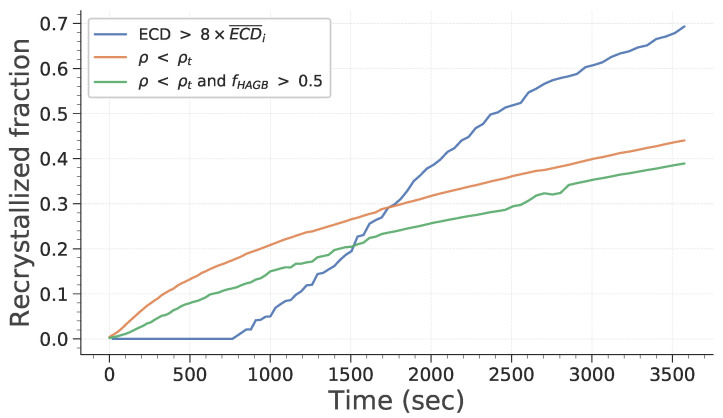
Evolution of recrystallized fractions with time.

Figure 10 illustrates how the recrystallized surface fraction evolves during simulation, for the first simulation case described in Section 3.1.3. As one could have expected, the recrystallized fraction behaves differently depending on its definition. Consequently, ensuring that the criteria used to discriminate recrystallized grains from experimental and simulation data are consistent is critical.

### 3.2. Modeling of CDRX and PDRX

Let us now examine simulations of CDRX and PDRX. To present the abilities of the model and evaluate the influence of subgrain formation rules, the results obtained with four different test cases are described below. All of them consider initial microstructures respecting the same grain size and hardening coefficient distributions. They include approximately 300 grains. The initial number of grains is taken to be low because it will increase substantially during deformation. Material parameters have been estimated based on experimental results obtained from conducting a thermomechanical testing campaign associated with extensive EBSD characterization. A detailed article will be dedicated to the presentation of these results and the comparison with some simulation results acquired using this numerical framework. The thermomechanical conditions corresponding to these simulations are the following: T = 650 ${}^{{}^{\circ}}$C $;\;\dot{\varepsilon }=1.0\;s^{-1};\;\varepsilon_{f}=1.35$. They are representative of the conditions experienced by a billet during the hot extrusion process. Additional details about the manufacture of zirconium alloys can be found in reference [37]. The four test cases differ with the GB properties and the restrictions set for formation of subgrains. In all of the cases except the last ones, subgrains cannot be placed too close to grain boundaries to avoid topological events that are not seen experimentally such as bulging boundaries. They are defined as followed:

(a) The number of subgrains that are formed at each deformation increment is computed individually per grain/subgrain. GB energy is described by the RS equation and GB mobility is isotropic.
(b) The number of subgrains that are formed at each deformation increment is computed for the whole domain. Then, new subgrains are positioned randomly within the RVE. GB energy is described by RS Equation (Equation (15)) and GB mobility is isotropic.
(c) The number of subgrains that are formed at each deformation increment is computed individually per grain/subgrain. GB energy is described by RS Equation (Equation (15)) and GB mobility is heterogeneous and computed using Equation (16).
(d) This last case respects the same rules than the first one, except that new subgrains can be placed on pre-existing grain boundaries.

Based on the discussion presented previously (see Section 3.1.4) and to be as close as possible to the criterion used commonly when working with experimental data, it has been decided to define as recrystallized the grains that fulfill the two following criteria:

- $\rho \leq \rho_{th}=1.0\times 10^{14}$ m${}^{-2}$,
- only grains, i.e., entities bounded by at least 50% of HAGB, are included in the measure of recrystallized grains.

Setting threshold values for these two variables is performed arbitrarily using experimental data.

#### 3.2.1. Evolution of Main Microstructure Descriptors during CDRX and PDRX

**Figure 11:**
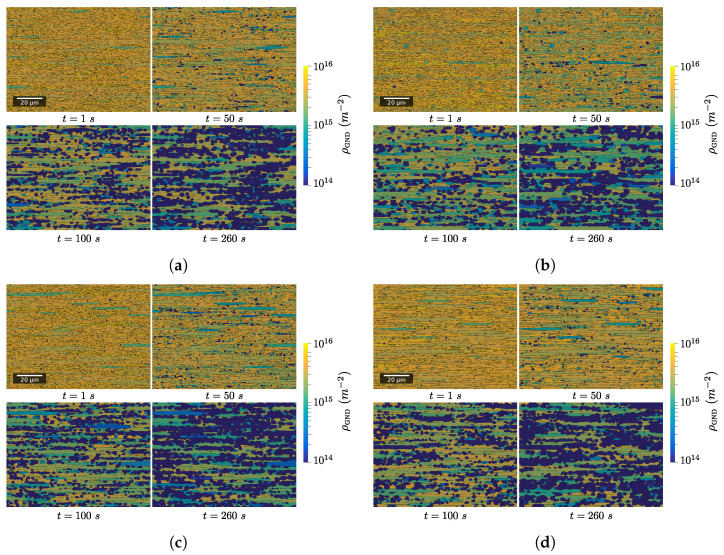
Evolution of digital microstructures with time for the four different test cases. (**a**) Local nucleation—$\gamma_{h}$. (**b**) Global nucleation—$\gamma_{h}$. (**c**) Local nucleation—$\gamma_{h},\;M_{h}$. (**d**) Local nucleation—$\gamma_{h},\;d_{NewLAGB}^{GB}=0$.

**Figure 12:**
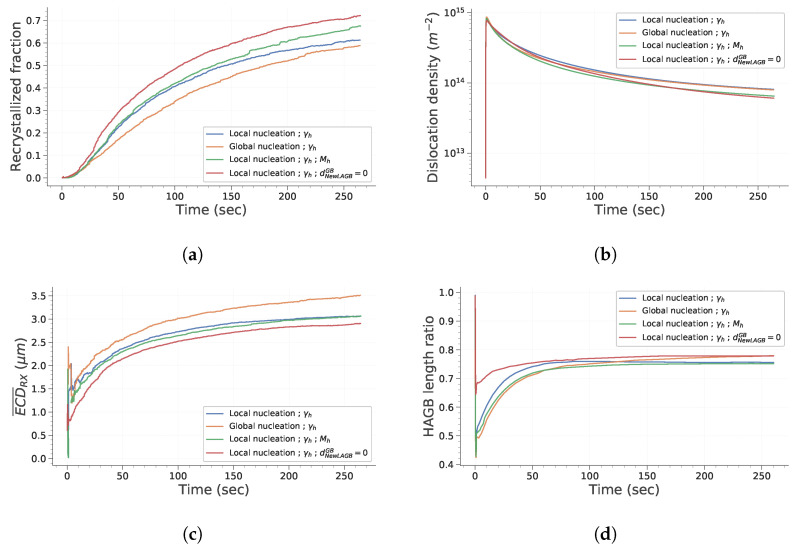
Main microstructural features’ evolution with time. (**a**) Recrystallized surface fraction. (**b**) GND density. (**c**) Mean recrystallized grain size. (**d**) HAGB length ratio.

Figure 11 presents the evolution of digital microstructures for the four test cases after a deformation of 1.35 and a subsequent holding at temperature (hundreds of seconds at 650 ${}^{{}^{\circ}}$C). Figure 12 presents the evolution of the main microstructural descriptors with time. First, one can see that the recrystallized fraction is null during the deformation process and starts to increase significantly only after tens of seconds of holding at temperature. This can be explained easily by analyzing the criteria defined previously for discrimination of recrystallized grains in regard to the mechanisms introduced in the numerical framework. First, only subgrains are formed during deformation due to recovery of a certain fraction of dislocations. These objects do not fulfill the second criterion. Then, under subsequent deformation, their misorientation increases as prescribed by Equation (14). However, at the same time, their internal dislocation density increases. Therefore, with the material parameters considered here, new subgrains see their dislocation density exceed the threshold value before they have time to transform into grains. Then, under holding at temperature, grains and subgrains that have an energetic advantage over the surrounding grains start to grow. Their dislocation density is progressively lowered. They are also likely to encounter new grains which generally lead to an increase in the bounding HAGB fraction. Consequently, more and more candidates are meeting both of the criteria and are flagged as recrystallized. Finally, evolution of average recrystallized grain size presents a sigmoidal shape, except for the first instants where there are abrupt and erratic variations. This is due to the small number of grains considered as recrystallized at those times. Finally, it is interesting to discuss how the HAGB length ratio evolves during the whole process (Figure 12). First, it decreases quickly with strain due to the fact that some new subgrains form. Then, once deformation is stopped, it increases by approximately 10% instantaneously. One reason for that observation is that the hardening parameter, $K_{1}$, attributed to each subgrain is taken from a distribution. Therefore, some subgrains have a higher hardening parameters than the parent grains and disappear as soon as their dislocation density is higher than the one of the parent grain. This is not visible during deformation because the formation of new subgrains is much more significant. Secondly, it is noteworthy to mention the rapid stagnation of the HAGB length ratio. This would mean that grains and subgrains that have a lower dislocation density grow with similar rates. This makes sense because the driving pressure induced by interface energy is negligible compared to the driving pressure linked to differences in the deformation stored energy.

Considering the differences between the results obtained with the four test cases, it appears that setting GB mobility as heterogeneous does not significantly impact the statistical descriptors presented here. One could notice that introducing subgrains over the whole domain globally affect recrystallized fraction at intermediate times and induces an increase in the average recrystallized grain size by approximately 15%. This difference in recrystallized fraction can be explained by the fact that the main driving pressure for the growth of recrystallized grains is the difference in stored energy between those grains and the surrounding grains. Therefore, if the quantity of interfaces created during deformation is individualized per grain, one can deduce from Equation (13) that more subgrains will be placed in zones with high dislocation density. Thus, the kinetic of recrystallization will be higher for intermediate holding times.

An explanation for the difference in the average recrystallized grain size can be found by investigating the neighbors of viable subgrains. When nucleation is set globally, viable subgrains that effectively form recrystallized grains can grow much significantly before encountering other recrystallized grains. Finally, regarding the last test case, allowing new subgrains to form at GB leads to a 10% higher recrystallized fraction. The reason behind that difference is that subgrains that form over pre-existing GB have already a fraction of their boundary as HAGB.

#### 3.2.2. Evolution of the Subgrain Network during Deformation

**Figure 13:**
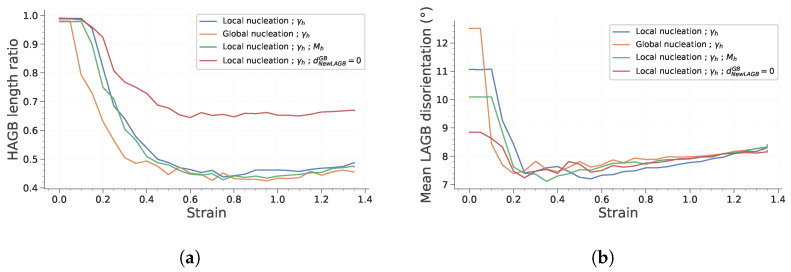
Main microstructural feature evolution with strain during deformation. (**a**) HAGB length ratio. (**b**) Mean LAGB disorientation.

The evolution of the HAGB length ratio and the mean LAGB disorientation with strain are presented in Figure 13. Focusing on the HAGB length ratio first, it is interesting to note that it reaches a steady state after a strain of 0.6. This is consistent with some experimental observations reported by Chauvy et al. [38]. As discussed in the previous section, some subgrains disappear during deformation because they harden too much to stay viable. The average LAGB disorientation increases by approximately $1^{{}^{\circ}}$ during deformation. This increase remains low because the disappearance and formation of subgrains impact significantly the subgrain population.

**Figure 14:**
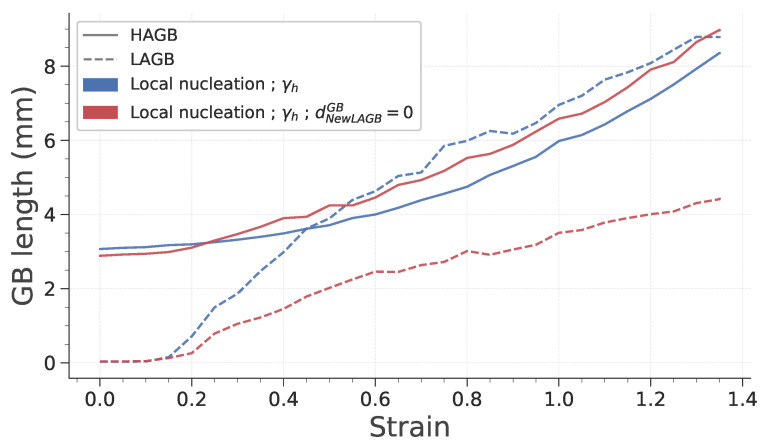
Evolution of LAGB and HAGB length with strain during deformation.

Finally, the last point worth mentioning is that the only case that exhibits different results regarding those two features is the last one, in which subgrains can form onto the GB. To investigate the reason behind this difference in HAGB length ratio, the evolution of LAGB and HAGB length with strain is plotted in Figure 14. It is interesting to note that both cases exhibit comparable HAGB length evolution. However, it appears that the reference case in which subgrains form at a given distance from GB, the increase in LAGB length is much higher. This is totally consistent, because the formation of subgrains over HAGB gives rise to a lower quantity of LAGB and to the disappearance of HAGB.

## 4. Conclusions

The present study has detailed the current abilities of the proposed numerical framework to predict the evolution of subgrain structures by GG, CDRX, and PDRX phenomena. The results concerning GG of a fully substructured microstructure have shone a light on:

- The influence of the microstructure topology;
- The significance of the definition of LAGB and subgrain properties. Indeed, it appeared that the strategy adopted by the microstructure to reduce the total system energy is different depending on those parameters. The observations have led to the deduction that heterogeneous mobility encourages a temporary increase in HAGB length to accelerate the general energy decrease. They also illustrated that preferential subgrain growth is much more significant if subgrains all have initially the same size;
- The fact that internal dislocation density has a non-negligible impact.

The results regarding the simulations of CDRX and PDRX have illustrated that this simulation environment coupled to the Gourdet–Montheillet model provides realistic and consistent results. They also show that the model is flexible enough to assess the impact of several hypotheses and assumptions.

The results of the present CDRX model now need to be confronted to experimental results to assess to which extent they are able to predict them. This will be presented in an upcoming article regarding microstructure evolution of zircaloy-4 in hot forming conditions. Moreover, the present CDRX and PDRX model could still be extended by being applied to other materials which exhibit different microstructure features due to the same CDRX mechanisms.

## Figures and Tables

**Figure A1:**
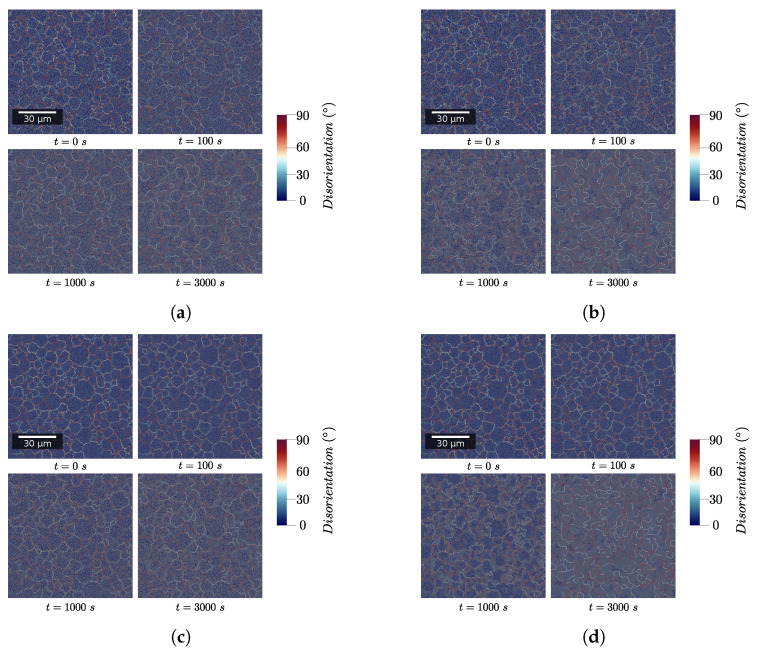
Evolution of GB network with time for the four test cases. (**a**) Experimental subgrain size distribution—$\gamma_{h}$. (**b**) Experimental subgrain size distribution—$\gamma_{h},\;M_{h}$. (**c**) Uniform subgrain size distribution—$\gamma_{h}$. (**d**) Uniform subgrain size distribution—$\gamma_{h},\;M_{h}$.

## Data Availability

The data necessary to reproduce these findings are available from the corresponding author on request.
